# The impact of Human Papilloma Virus status on the prediction of head and neck cancer chemoradiotherapy outcomes using the pre-treatment apparent diffusion coefficient

**DOI:** 10.1259/bjr.20210333

**Published:** 2021-12-20

**Authors:** Steve Connor, Mustafa Anjari, Christian Burd, Amrita Guha, Mary Lei, Teresa Guerrero-Urbano, Irumee Pai, Paul Bassett, Vicky Goh

**Affiliations:** 1 School of Biomedical Engineering and Imaging Sciences, St Thomas’ Hospital, King’s College, London, United Kingdom; 2 Department of Neuroradiology, King’s College Hospital, London, United Kingdom; 3 Department of Radiology, Guy’s Hospital, London, United Kingdom; 4 Department of Radio-diagnosis, Tata Memorial Hospital, Parel, Homi Bhabha National Institute, Mumbai, India; 5 Department of Oncology, Guy’s Hospital, London, United Kingdom; 6 Department of Ear, Nose and Throat Surgery, Guy’s and St Thomas’ Hospital, London, United Kingdom; 7 Freelance Medical Statistician, London, United Kingdom

## Abstract

**Objective:**

To determine the impact of Human Papilloma Virus (HPV) oropharyngeal cancer (OPC) status on the prediction of head and neck squamous cell cancer (HNSCC) chemoradiotherapy (CRT) outcomes with pre-treatment quantitative diffusion-weighted magnetic resonance imaging (DW-MRI).

**Methods:**

Following ethical approval, 65 participants (53 male, age 59.9 ± 7.86) underwent pre-treatment DW-MRI in this prospective cohort observational study. There were 46 HPV OPC and 19 other HNSCC cases with Stage III/IV HNSCC. Regions of interest (ROIs) (volume, largest area, core) at the primary tumour (*n* = 57) and largest pathological node (*n* = 59) were placed to analyse ADC_mean_ and ADC_min_. Unpaired *t*-test or Mann–Whitney test evaluated the impact of HPV OPC status and clinical parameters on their prediction of post-CRT 2 year locoregional and disease-free survival (LRFS and DFS). Multivariate logistic regression compared significant variables with 2 year outcomes.

**Results:**

On univariate analysis of all participants, the primary tumour area ADC_mean_ was predictive of 2 year LRFS (*p* = 0.04). However, only the HPV OPC diagnosis (LFRS *p* = 0.03; DFS *p* = 0.02) predicted outcomes on multivariate analysis. None of the pre-treatment ADC values were predictive of 2 year DFS in the HPV OPC subgroup (*p* = 0.21–0.68). Amongst participants without 2 year disease-free survival, HPV-OPC was found to have much lower primary tumour ADC_mean_ values than other HNSCC.

**Conclusion:**

Knowledge of HPV OPC status is required in order to determine the impact of the pre-treatment ADC values on post-CRT outcomes in HNSCC.

**Advances in knowledge:**

Pre-treatment ADC_mean_ and ADC_min_ values acquired using different ROI methods are not predictive of 2 year survival outcomes in HPV OPC.

## Introduction

Head and neck squamous cell cancer (HNSCC) is the seventh commonest cancer worldwide.^1^ Concomitant chemoradiotherapy (CRT) is the standard of care for advanced locoregional disease at most head and neck tumour sites. However, treatment fails in more than 30% of advanced stage tumours, and salvage surgery for locoregional recurrence has a potentially poor outcome.^2^ Identifying tumours that may not respond to CRT provides prognostic information, guides more proactive surveillance, and prompts more intensive or modified treatment plans such as radiotherapy dose escalation or surgery.

One potential imaging biomarker is the pre-treatment tumour apparent diffusion coefficient (ADC) on diffusion-weighted MRI (DW-MRI). A number of studies have assessed the value of pre-treatment ADC_mean_ in predicting HNSCC treatment response.^3–25^ Whilst many have demonstrated that that a high pre-treatment ADC_mean_ predicts a poor outcome following CRT,^6–8,11,12,15,18–21^ there are conflicting results.^3–5,9,10,13,14,16,17,22–25^

Human Papilloma Virus (HPV) oropharyngeal cancer (OPC) status may be an important confounding factor in these studies, since its unique histopathological characteristics^26^ may both influence ADC_mean_ measures^27–30^ and clinical outcomes. HPV OPC is increasing in incidence and now accounts for 70–80% of OPC in the United States and Western Europe.^31^ Whilst the HPV OPC status is rarely documented in studies of pre-treatment ADC values in predicting HNSCC outcomes, recent evidence has suggested it to be a potentially important factor.^4,14,15,24^

Our primary objective was to determine the impact of HPV OPC status on the ability of pre-treatment DW-MRI ADC values to predict 2 year survival outcomes in Stage 3 and 4 HNSCC following CRT.

## Methods

### Participants

Participants were recruited for a prospective single centre cohort observational study between 2014 and 2017 (http://www.controlled-trials.com/ISRCTN58327080) following Research Ethics Committee approval (REC reference 13/LO/1876) and informed consent.

Participants were eligible if there was histologically confirmed Stage 3 or 4 primary HNSCC without distant metastases, an axial section delineating an 1 cm^2^ area of measurable primary tumour and/or pathological nodal tumour on standard clinicoradiological staging, and curative primary CRT was planned. Data collection included demographic and clinical information (age, gender, tumour subsite and tumour stage). Exclusion criteria were prior chemoradiotherapy, an ECOG performance status >2, lack of capacity to understand the patient information sheet or inability to provide informed consent, and known allergy to gadolinium-based contrast medium or eGFR <30 ml/min.

Sample size calculation was based on 70% of patients demonstrating 2 year disease free survival (DFS). Based on previous reports,^5,7,11,12,19^ the ADC measurements were predicted to have a standard deviation of 40 and to demonstrate a difference of 50 × 10^−6^ mm^2^/s between participants with and without 2 year survival outcomes. Using a 5% significance level and 80% power, it was calculated that at least 40 subjects would be required for the study without any accounting for loss of data.

#### HPV status, biopsies and treatment

HPV status was analysed for all OPC as per standard of care. Non-OPC HNSCC was not routinely tested for HPV status, according to international guidelines.^32^ HPV status was evaluated with p16 testing using an immune-stain or high-risk HPV DNA testing using *in situ* hybridisation. Biopsies at diagnosis were obtained from the primary tumour (*n* = 56), lymph node (*n* = 7) or both (*n* = 2). Intensity modulated radiotherapy (IMRT) was delivered as per the standard of care which was 7 Gy in 35 fractions; 2 Gy per fraction delivered once daily, 5 days a week. Concomitant intravenous cisplatin at a dose of 35 mg/m^2^ every 7 days, starting on Day 1 of radiotherapy, was administered in all patients with adequate GFR and no contraindications (*n* = 47) with carboplatin (*n* = 16) or radiotherapy alone (*n* = 2) being used in the remaining participants.

The cohort was subsequently divided into HPV OPC and all other HNSCC (including HPV negative OPC) for analysis as in previous studies.^4,13^ This approach was adopted, since HPV OPC is a distinct entity with unique histopathology and favourable treatment outcomes relative to all other HNSCC and the prognostic impact of p16 positive status is restricted to OPC only.^33,34^

### MR imaging

#### Protocol and technique

Participants underwent 1.5 Tesla MRI (Magnetom Aera, Siemens Healthcare, Erlangen, Germany) using a surface phased array 20 channel neck coil. The routine MRI protocol (Table 1) was supplemented by a research axial echo planar diffusion-weighted sequence with multiple b-values (0, 50, 100, 800 and 1500 s/mm^2^) and the following scan parameters: repetition time 5900 ms, echo time 60 ms, two signal averages, FOV 240 x 240 mm, slice thickness 4 mm with a 0.5 mm slice gap. Mono-exponential ADC maps were calculated from the *b* = 100 and *b* = 800 values.

**Table 1.** MRI protocol

	Plane	Slice thickness/ gap (mm)	TR/TE	Field of view (mm)	Number of averages	Pixel bandwidth (Hz/pixel)	Flip angle (degrees)	Acquisition matrix
***T*_1_W**	axial	4/0	549/11	220 × 220	1	200	160	384 × 269
***T*_2_W**	axial	4/0	5830/102	220 × 220	1	190	150	384 × 346
***T*_1_W fat saturated -DIXON post gadolinium**	axial	4/0	566/11	220 × 220	1	330	145	320 × 224
**STIR**	coronal	3/0.3	3000/35 TI 140	260 × 260	1	220	160	320 × 224
***T*_1_W fat sat-DIXON post-gadolinium**	coronal	3/0.3	708/10	280 × 280	1	340	145	320 × 320

#### MRI processing and analysis

The ROIs were placed by a radiologist (MA, 3 years of experience) under the supervision of another radiologist (SC, 21 years of experience). The first 22 primary tumours and 24 nodes were also independently analysed by another radiologist (AG, 5 years of experience) to assess interobserver agreement.

**Figure 1.**
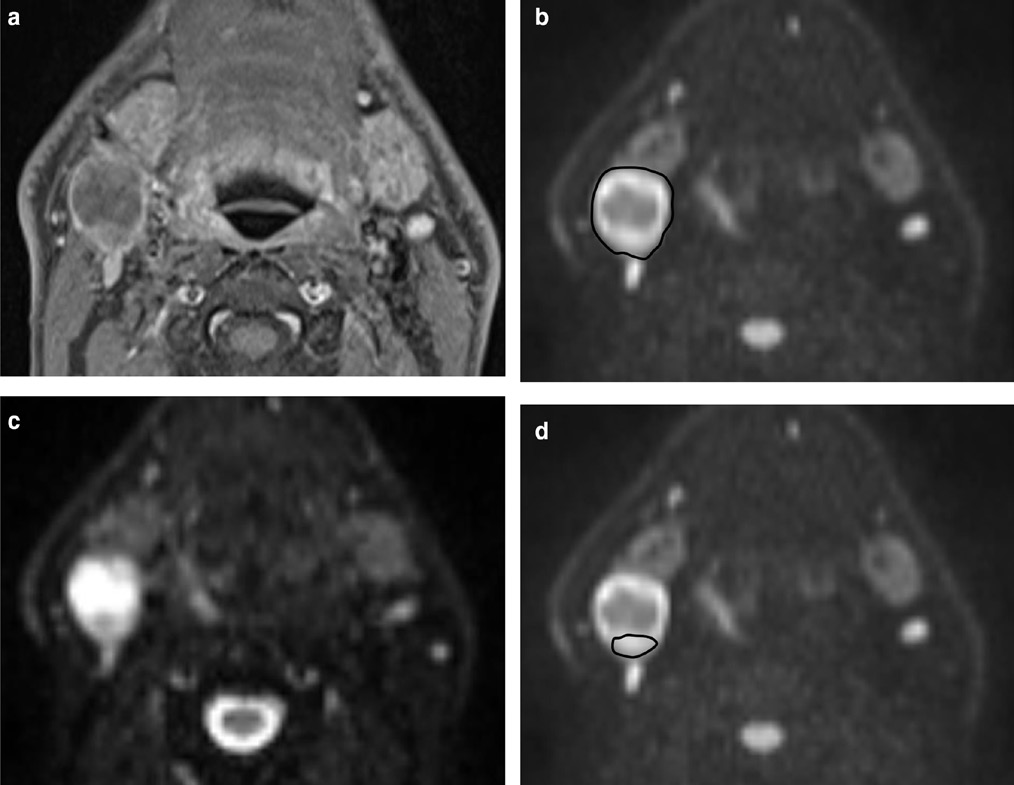
A HPV positive oropharyngeal cancer patient with a largely cystic right level two lymph node. (**a**) *T*_1_W post-gadolinium axial image b) *b* = 0 s/mm^2^ map from DW-MRI c) *b* = 800 s/mm^2^ map from DW-MRI indicating aROI as the whole of the lymph node outline and d) *b* = 800 s/mm^2^ map from DW-MRI indicating rROI as the component of the lesion returning intermediate signal on the *b* = 0 map and with enhancement on the *T*_1_W post gadolinium axial image. DW-MRI, diffusion-weighted MRI; HPV, human papilloma virus; ROI, region of interest.

**Figure 2.**
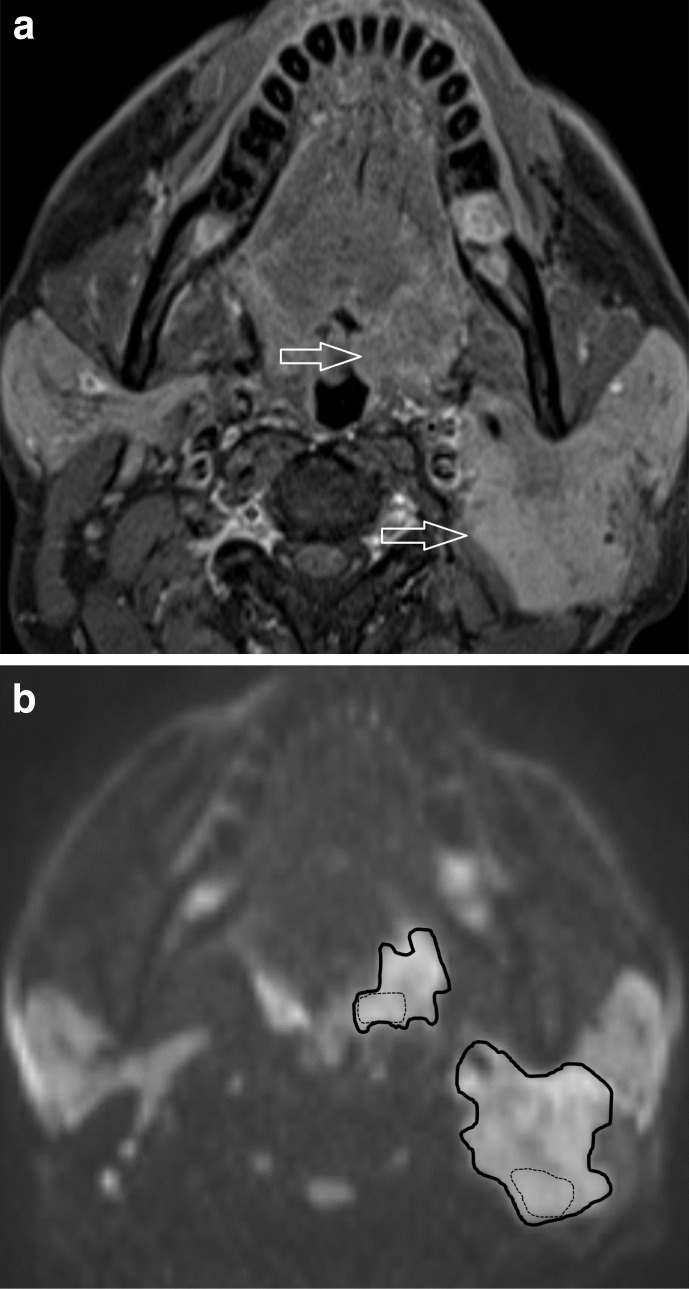
A HPV negative oropharyngeal cancer patient with a left palatine tonsillar cancer and left level two necrotic lymph node. (**a**) T1w post gadolinium axial image demonstrates the left palatine tonsillar tumour and the left level two lymph node (arrows). (**b**) *b* = 800 s/mm^2^ map from DW-MRI indicating aROIs as the continuous bold lines and rROIs as the dotted lines. DW-MRI, diffusion-weighted MRI; HPV, human papilloma virus; ROI, region of interest.

Free hand ROIs were placed using OsiriX v. 8.0.2, open source Mac-based medical image processing software with the images magnified to a standard 300%. They were defined on the DWI *b* = 800 s/mm^2^ map, but with access to the post-gadolinium fat saturated T1 axial sequence. Three separate ROIs were placed individually within the primary tumour and/or largest pathologically appearing lymph node (Figures 1 and 2):

- Volumetric ROI (vROI) placed on multiple sections to encompass the whole lesion volume.
- Area ROI (aROI) placed around the largest single cross-sectional area of the lesion.
- Representative area ROI (rROI) placed on the largest cross-sectional area in the core of the lesion. This focused on an area of increased DWI signal on the *b* = 800 s/mm^2^ map but excluded any areas of necrosis defined by cross-referencing to areas of either high signal on the *b* = 0 map or absence of gadolinium enhancement.

Areas of peritumoural inflammation characterised by high signal on the *b* = 0 map and avid gadolinium enhancement were avoided on all ROIs. An ADC map was generated from the b100 and b800 s/mm^2^ images to mitigate the perfusion effects. ADC_mean_ and standard deviation were recorded with ADC_min_ calculated as ADC_mean_ – one standard deviation (SD).^21^ The vROI was also used to define the volume of the primary tumour and/or largest lymph node using the summation of areas technique. A ROI was also placed within the cervical spinal cord on the ADC map, and ADC_mean_ was calculated as a reference for any non-patient related variability or artefact.

### Treatment outcome

Outcome evaluation comprised clinical assessment at 1 year and 2 years following completion of treatment. Treatment failure was determined by cytological or histological confirmation (biopsy or resection) or by serial progression on imaging follow-up. The 2 year locoregional recurrence free survival (LRFS) and 2 year disease free survival (DFS) were recorded according to the status at 2 years following completion of treatment.

A standard of care 12 week post-treatment positron emission tomography computed tomography (PET CT) study was used to guide clinical management. A quantitative evaluation of the post-treatment ^18^F-FDG PET-CT was also obtained. This comprised a 6 mm diameter volume of interest (VOI) being placed at the site of most intense FDG uptake within either the primary lesion and/or the largest lymph node. If there was reduced uptake on the post-treatment images relative to background, a 6 mm VOI was placed at the same site as the post-treatment MRI ROI. If necrosis was identified within a lesion, the area of necrosis was excluded. The SUV_max_ was calculated with semi-automated software on a Hermes workstation (Hermes Gold 3, Stockholm).

### Statistical analysis

Statistical analysis was performed using Stata (v. 15.1) with a *p* value of < 0.05 being considered statistically significant.

Descriptive statistics summarised the patient demographics, HPV status, tumour site and staging.

ADC_mean_ and ADC_min_ values obtained using the different ROI methods at both primary tumour and nodal locations were analysed for all participants, and for HPV OPC and other HNSCC subgroups. ADC values were compared between HPV OPC and other HNSCC participants.

ADC values, HPV OPC status and clinical variables (tumour site and staging, patient gender and age) were compared with the dichotomised 2 year outcomes of DFS and LRFS. Any variables associated with 2 year survival outcomes (*p* < 0.2) were used as independent variables in the multivariate analysis.

Continuous variables were compared using the unpaired *t*-test if normally distributed, and the Mann–Whitney test if not normally distributed. Categorical variables were compared between the two groups using the χ^2^ test or Fisher’s exact test.

Pearson’s correlation coefficient was used to measure the relationship between pre-treatment ADC_mean_ or ADC_min_ values and the 12 week post-CRT ^18^F-FDG PET-CT SUV_max_for all participants.

The Intraclass correlation coefficients (ICCs) for each of the vROI, aROI and rROI at primary tumour and nodal locations were evaluated in the sample analysed by two observers.

## Results

### Participant and tumour characteristics

**Figure 3.**
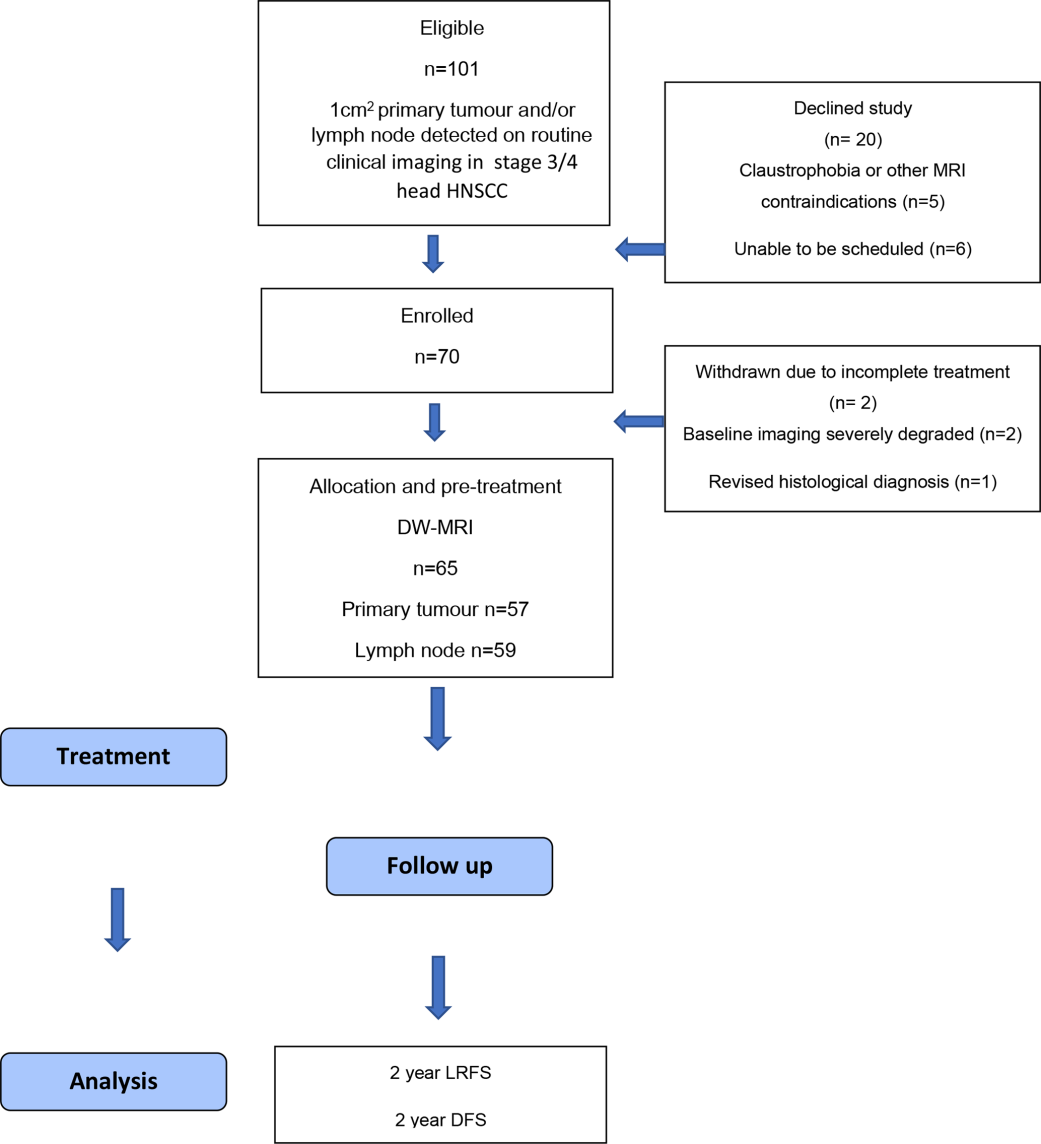
Participant flow chart.

The participant flowchart is summarised in Figure 3.

There were 101 patients eligible according to study criteria with 70 patients enrolled, but five were subsequently withdrawn. Of the 65 participants (53 male, 12 female, mean age 59.9 ± 7.86), there were 54 (83%) Stage 4 and 11 (17%) Stage 3 disease. Participant characteristics including HPV status, primary site and staging (according to International Union Against Cancer seventh edition of the TNM classificiation) and are summarised in Table 2. There were 46/65 patients with HPV OPC and 19/65 with other HNSCC.

**Table 2.** Primary tumour site and subsite, TN staging (TNM 7) and HPV status for the 65 patients

	Subsite			T stage					N stage					HPV status		
				**T0**	**T1**	**T2**	**T3**	**T4**	**NO**	**N1**	**N2A**	**N2B**	**N2C**	**+ve**	-**ve**	**Not tested**
**Oro** **pharynx** **(*n* = 49)**	**Tongue** **base (29)**	**Tonsil** **(19)**	**Soft palate (1)**	1	7	18	7	16	3	4	3	29	10	46	3	0
**Larynx** **(*n* = 10)**	**Supra** **glottic** **(7)**	**Trans glottic** **(3)**					8	2	0	5	3	1	1	0	2	8
**Hypopharynx** **(*n* = 6)**	**Piriform fossa** **(6)**					3	2	1				4	2	0	0	6

HPV, Human Papilloma Virus.

At 2 year follow-up, there were a total of 10 participants with recurrence (isolated nodal recurrence *n* = 4; nodal, primary and distal metastatic recurrence *n* = 1; isolated primary recurrence *n* = 1; distal metastases alone *n* = 4). Five participants with nodal recurrence underwent salvage neck dissection. Median time to recurrence was 185.5 [108,263] days post treatment. There were 55/65 patients with 2 year DFS and 59/65 patients with 2 year LRFS. The subsequent median follow-up was 4.1 [3.05,5.0] years post-treatment and there were no other cases of progressive disease. There were 57/65 patients who attended for the 12 week post-CRT ^18^F-FDG PET-CT study.

ROI was delineated at the primary tumour (*n* = 6), the largest lymph node (*n* = 8) or both locations (*n* = 51). Median area of aROI was 166 [88-346] mm^2^ for primary tumour and 281 [171-415] mm^2^ for the lymph nodes. Median volume of vROI was 8908 [3704,12664] mm^3^ for primary tumour and 4220 [2130,8270] mm^3^ for the lymph nodes. The mean cervical spinal cord ROI ADC_mean_ was 1004 ± 79 x 10^−6^ mm^2^/s.

### Analysis of ADC values and 2-year outcomes

The ADC_mean_ and ADC_min_ values obtained at the primary tumour site were significantly lower in HPV OPC participants than other HNSCC participants for all ROI analyses (*p* < 0.001). However, the the nodal ADC values did not differ between HPV OPC and other HNSCC subgroups (*p* = 0.3–0.61).

On univariate analysis of ADC parameters and 2 year survival outcomes for all participants, the only statistically significant association was that demonstrated between the primary tumour aROI ADC_mean_ and 2 year LRFS (*p* = 0.04, Figure 3) (Table 3). HPV OPC status was also predictive of 2 year LRFS (*p* = 0.03) and DFS (*p* = 0.02) on univariate analysis (Table 4).

**Table 3.** Comparison of primary tumour and nodal ADC_mean_ and ADC_min_ values for vROI, aROI, rROI between participants with and without 2 year DFS and 2 year-LRFS: all participants

*Variable*	*No 2 year DFS* *ADC (10^−6^ mm^2^/s)*		*2 year DFS* *ADC (10^−6^ mm^2^/s)*		*p-value*
	*n*		*n*		
Lymph node vROI ADC_mean_	8	964 [936, 1189]	51	1033 [891, 1258]	0.88
Lymph node vROI ADC_min_	8	744 ± 121	51	820 ± 206	0.32
Lymph node aROI ADC_mean_	8	1014 [1040, 1221]	51	1001 [862, 1175]	0.88
Lymph node aROI ADC_min_	8	800 [704, 843]	51	814 [668, 926]	0.60
Lymph node rROI ADC_mean_	8	930 ± 94	51	955 ± 182	0.70
Lymph node rROI ADC_min_	8	771 ± 138	51	808 ± 167	0.55
Primary tumour vROI ADC_mean_	10	1085 ± 259	47	1018 ± 183	0.33
Primary tumour vROI ADC_min_	10	823 ± 203	47	789 ± 162	0.57
Primary tumour aROI ADC_mean_	10	1079 ± 263	47	978 ± 190	0.16
Primary tumour aROI ADC_min_	10	849 ± 193	47	776 ± 184	0.26
Primary tumour rROI ADC_mean_	10	958 [808, 1201]	47	863 [779, 996]	0.32
Primary tumour rROI ADC_min_	10	836 ± 216	47	778 ± 182	0.38

*Variable*	*No 2 year LFRS* *ADC (10^−6^ mm^2^/s)*		*2 year LFRS* *ADC (10^−6^ mm^2^/s)*		*p-value*
	*n*		*n*		
Lymph node vROI ADC_mean_	5	1175 [966, 1202]	54	1000 [898, 1168]	0.64
Lymph node vROI ADC_min_	5	727 ± 151	54	817 ± 201	0.33
Lymph node aROI ADC_mean_	5	1113 [1037, 1128]	54	994 [875, 1153]	0.74
Lymph node aROI ADC_min_	5	783 [669, 829]	54	816 [687, 917]	0.48
Lymph node rROI ADC_mean_	5	948 ± 115	54	952 ± 177	0.96
Lymph node rROI ADC_min_	5	749 ± 173	54	808 ± 162	0.44
Primary tumour vROI ADC_mean_	6	1164 ± 293	51	1014 ± 181	0.08
Primary tumour vROI ADC_min_	6	862 ± 235	51	788 ± 160	0.31
Primary tumour aROI ADC_mean_	**6**	**1158 ± 301**	**51**	**977 ± 187**	0.04*
Primary tumour aROI ADC_min_	6	888 ± 229	51	777 ± 180	0.17
					
Primary tumour rROI ADC_mean_	6	1011 [808, 1201]	51	863 [779, 996]	0.25
Primary tumour rROI ADC_min_	6	851 ± 242	51	781 ± 182	0.39

ADC, apparent diffusion coefficient; DFS, disease free survival; LRFS, locoregional and disease-free survival; ROI, region of interest; VOI, volume of interest.

Summary statistics are: number (percentage), mean ± standard deviation or median [interquartile range]

Continuous variables were compared using the independent t-test if normally distributed, and the Mann–Whitney test if not normally distributed*indicates statistically significant association.

**Table 4.** Comparison of patient and disease characteristics between participants with and without 2 year DFS and LRFS

*Variable*	*Category*	*No 2 year DFS*		*2 year DFS*		*p*-value
		*n*		*n*		
Tumour	Oropharynx	10	5 (50%)	55	44 (80%)	0.1
subsite	Larynx		3 (30%)		7 (13%)	
	Hypopharynx		2 (20%)		4 (7%)	
Tumour stage	Stage 3	10	1 (10%)	55	10 (18%)	1
	Stage 4		9 (90%)		45 (82%)	
Gender	Male	10	7 (70%)	55	46 (84%)	0.31
	Female		3 (30%)		9 (16%)	
Age	-	10	61.2 ± 8.6	55	59.7 ± 7.7	0.58
HPV OPC status	Negative	**10**	**6 (60%)**	**55**	**13 (24%)**	0.02*
	Positive		**4 (40%)**		**42 (76%)**	

*Variable*	*Category*	*No 2 year LRFS*		*2 year LRFS*		***p*** **-value**
		*n*		*n*		
Tumour	Oropharynx	**6**	**2 (33%)**	**59**	**47 (80%)**	0.02*
subsite	Larynx		2 (33%)		8 (14%)	
	Hypopharynx		2 (33%)		4 (7%)	
Tumour stage	3	6	1 (10%)	59	10 (17%)	1
	4		5 (83%)		49 (83%)	
Gender	Male	6	4 (67%)	59	49 (83%)	0.32
	Female		2 (33%)		10 (17%)	
Age	-	6	61.3 ± 9.4	59	59.8 ± 7.7	0.65
HPV OPC status	Negative	**6**	**4 (67%)**	**59**	**15 (25%)**	0.03*
	Positive		**2 (33%)**		**44 (75%)**	

HPV, Human Papilloma Virus; LRFS, locoregional and disease-free survival; OPC, oropharyngeal cancer.

Summary statistics: number (percentage), mean ± standard deviation when normally distributed and median [inter-quartile range] when not normally distributed

Continuous variables compared using the independent t-test if normally distributed, and the Mann-Whitney test if not normally distributed*indicates statistically significant association.

**Figure 4.**
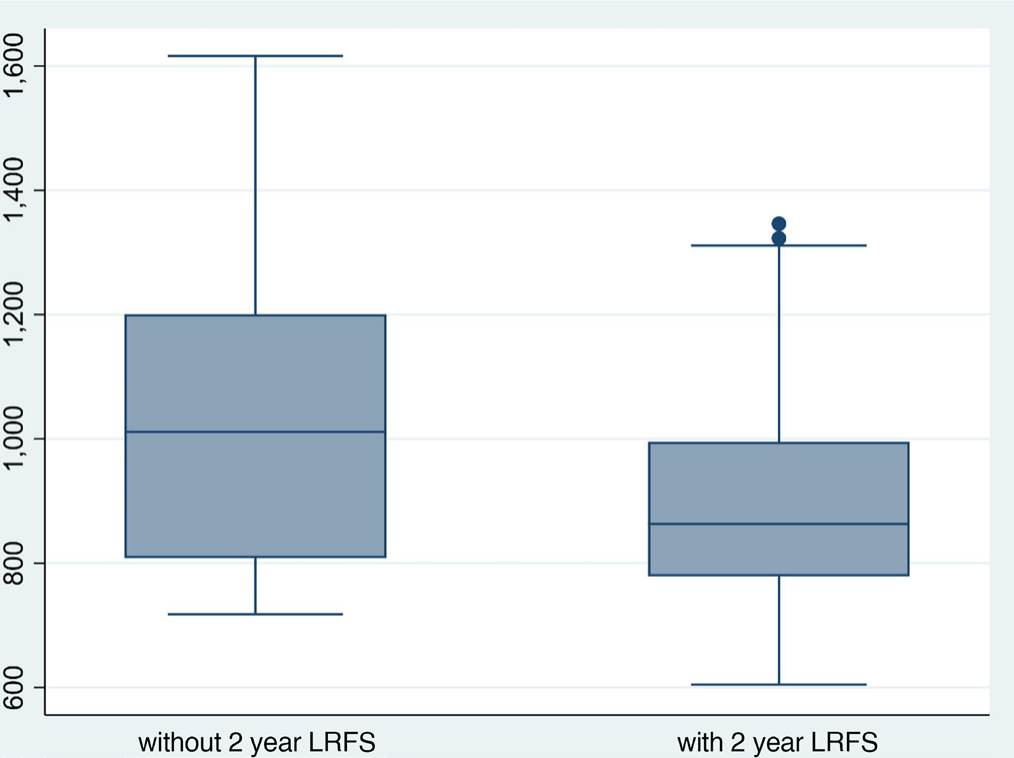
Primary tumour aROI ADC (10^−6^ mm^2^/s) in patients with and without 2 year LRFS. ADC< apparent diffusion coefficient; LRFS, locoregional and disease-free survival; ROI, region of interest.

On multivariate regression analysis, only HPV OPC status maintained a significant association with 2 year DFS (*p* = 0.03) and LRFS (*p* = 0.05) with odds ratio (95% CI) of 4.85 (1.18–19.8) for DFS and odds ratio (95% CI) of 5.87 (0.97–35) for LRFS (Figure 4). The primary tumour aROI ADC_mean_ was no longer able to predict 2 year LRFS.

The subgroup analysis did not demonstrate any association between HPV OPC ADC values and 2 year DFS (*p* = 0.21–0.68); however, due to the limited sample size 2 year LRFS analysis was not possible. Similarly, ADC values were unable to predict 2 year DFS in other HNSCC (*p* = 0.66–0.85), although the sample size was limited. In participants without 2 year DFS, the ADC values were markedly lower in HPV OPC compared to other HNSCC (Table 5). For instance ADC_mean_ at the primary tumour site was 863 ± 74 x 10^−6^ mm^2^/s for HPV OPC (*n* = 4) whilst 1138 ± 179 x 10^−6^ mm^2^/s for other HNSCC (*n* = 6), however, the sample size without 2 year DFS was too small for statistical comparison.

**Table 5.** Comparison of primary tumour and nodal ADC_mean_ and ADC_min_ values for vROI, aROI, rROI between participants with and without 2 year DFS : HPV OPC and other HNSCC

*Variable*	*No 2 year DFS* *ADC (10^−6^ mm^2^/s)*		*2 year DFS* *ADC (10^−6^ mm^2^/s)*		*p-value*
***Other HNSCC***	***n***		***n***		
Lymph node vROI ADC_mean_	4	1071 ± 263	10	1063 [1091,1358]	0.73
Lymph node vROI ADC_min_	4	923 ± 169	10	846 ± 206	0.77
Lymph node aROI ADC_mean_	4	1082 ± 289	10	1101 [962, 1375]	0.76
Lymph node aROI ADC_min_	4	934 ± 174	10	814 [668, 926]	0.71
Lymph node rROI ADC_mean_	4	990 [888, 1059]	10	955 ± 182	0.70
Lymph node rROI ADC_min_	4	919 ± 168	10	808 ± 167	0.85
Primary tumour vROI ADC_mean_	6	1128 ± 181	13	1018 ± 183	0.81
Primary tumour vROI ADC_min_	6	823 ± 203	13	789 ± 162	0.76
Primary tumour aROI ADC_mean_	6	1138 ± 179	13	978 ± 190	0.76
Primary tumour aROI ADC_min_	6	849 ± 193	13	776 ± 184	0.74
Primary tumour rROI ADC_mean_	6	1122 ± 224	13	863 [779, 996]	0.81
Primary tumour rROI ADC_min_	6	836 ± 216	13	778 ± 182	0.66

*Variable*	*No 2 year DFS* *ADC (10^−6^ mm^2^/s)*		*2 year DFS* *ADC (10^−6^ mm^2^/s)*		*p-value*
***HPV OPC***	***n***		***n***		
Lymph node vROI ADC_mean_	4	938 [872, 1098]	41	1002 [898, 1142]	0.50
Lymph node vROI ADC_min_	4	679 ± 99	41	814 ± 208	0.21
Lymph node aROI ADC_mean_	4	962 [839, 1052]	41	989 [875, 1150]	0.50
Lymph node aROI ADC_min_	4	761 [607, 820]	41	799[659, 900]	0.40
Lymph node rROI ADC_mean_	4	888 ± 114	41	943 ± 142	0.45
Lymph node rROI ADC_min_	4	803 ± 187	41	740 ± 136	0.33
Primary tumour vROI ADC_mean_	4	912 ± 80	34	960 ± 134	0.49
Primary tumour vROI ADC_min_	4	862 ± 235	34	740 ± 136	0.56
Primary tumour aROI ADC_mean_	4	863 ± 74	34	918 ± 137	0.45
Primary tumour aROI ADC_min_	4	691 ± 97	34	725 ± 157	0.68
Primary tumour rROI ADC_mean_	4	793 [737, 904]	34	806 [760, 939]	0.68
Primary tumour rROI ADC_min_	4	673 ± 112	34	735 ± 171	0.49

ADC, apparent diffusion coefficient; DFS, diesease free survival; HNSCC, head and neck squamous cell carcinoma; LRFS, locoregional and disease-free survival; OPC, oropharyngeal cancer; ROI, region of interest.

Summary statistics: number (percentage), mean ± standard deviation when normally distributed and median [inter-quartile range] when not normally distributed

There was no significant correlation (*p* < 0.05) demonstrated between the pre-treatment ADC_mean_ or ADC_min_ values and the 12 week post-CRT ^18^F-FDG PET-CT SUV_max_ when applying any of the different ROI methods at either primary tumour and nodal locations.

The ICCs for the sample of ROIs performed by two observers were 0.97 (vROI), 0.98 (aROI) and 0.98 (rROI) for the primary tumour and 0.98 (vROI), 0.98 (aROI) and 0.94 (rROI) for the lymph node locations.

## Discussion

This study provides data concerning the impact of HPV OPC status on the ability of pre-treatment ADC values to predict survival outcomes. On multivariate analysis, the HPV OPC status was the only independent variable predicting both 2 year DFS and LRFS, and the apparent association between the primary tumour aROI ADC_mean_ and 2 year LRFS detected on univariate analysis was no longer evident. The pre-treatment primary tumour ADC values were lower in HPV OPC than other HNSCC, both overall and in those without 2 year survival. In this study cohort, it was not possible to demonstrate any prognostic ability of pre-treatment ADC values, when stratified by HPV OPC status.

Many previous studies have shown that an increased pre-treatment ADC is associated CRT treatment failure in HNSCC.^6–8,11,12,15,18–21^ It has been proposed that this may relate to the histological findings of higher stromal content, lower cellularity and micronecrosis, all of which are known to be associated with greater treatment resistance. However, a broad range of ADC thresholds have been reported for the prediction of treatment response and some studies have shown no significant association between the ADC values and outcome.^3–5,9,10,13,16,22–25^ Furthermore, one study has revealed the conflicting finding of poorer outcomes in tumours with lower ADC values; a result possibly explained by an association with poorly differentiated HNSCC.^17^

Previous data also suggest that HPV OPC is associated with lower pre-treatment ADC values compared to other HNSCC, and this finding is corroborated by our data.^27–30^ Despite this, studies correlating ADC values with post-CRT survival outcomes generally comprise heterogenous cohorts and only a few provide data on HPV OPC status^15,17^ or analyse HPV OPC cohorts separately.^4,13,24,35,36^ There are only four studies which have considered HPV OPC status as a co-variate.^4,14,15,24^ Ravanelli et al^24^ conducted a retrospective study comparing pre-treatment ADC_mean_ at the primary oropharyngeal tumour site in both HPV positive and negative OPC. In keeping with the results of this study, they also demonstrated that primary tumour aROI ADC_mean_ predicted survival outcomes on univariate analysis but not on multivariate analysis.^24^ Cao et al^4^ and Martens et al^14,15^ adopted a similar approach to our study and compared HPV OPC with other HNSCC at both primary tumour and nodal sites. Cao et al^4^ concluded that pre-treatment ADC values in HPV OPC were not associated with treatment failure; however, unlike the present study, they did demonstrate a correlation in their larger cohort of other HNSCC. Martens et al^14,15^ performed both retrospective and prospective studies and did not find pre-treatment ADC_mean_ to be predictive of survival outcomes in a multivariate analysis which included HPV status. The current study adds to the existing literature by analysing pre-treatment ADC values in HPV OPC and other HNSCC cohorts with a more comprehensive methodology, which included multiple approaches to contouring ROIs and the application of both ADC_mean_ and ADC_min_.

The favourable treatment response of low ADC tumours in previous studies^6–8,11,12,15,18–21^ has been considered counterintuitive, since low ADC values are generally found in poorly differentiated HNSCC, which would be expected to have a poorer prognosis. However, it could be postulated that the association of lower ADC values with HPV OPC status is primarily responsible for the apparent improvement in outcomes. Such a hypothesis is supported by our data, since multivariate analysis indicated that primary tumour ADC_mean_ was no longer of prognostic significance, and it was only HPV OPC status that was an independent factor associated with 2 year survival.

An awareness of HPV OPC status is clearly of importance in order to interpret the significance of pre-treatment ADC values. The markedly lower primary tumour ADC values in HPV OPC non-survivors compared to other HNSCC non-survivors would preclude the prediction of treatment failure without information on HPV OPC status. Similarly, the wide range of the primary tumour pre-treatment ADC_mean_ values in participants that did respond to treatment in HPV OPC (*e.g.* aROI 686–1684 x 10^−6^ mm^2^/s) would also limit the prediction on an individual basis without this knowledge.

Another factor to consider in the interpretation of previous literature, is that different methods of contouring ROIs have been variably applied. Few studies have applied multiple methods in the same cohort and assessed the impact on the prognostic value of measurements obtained. Whilst smaller ROIs are potentially less representative, they may benefit from the ability to exclude macroscopic necrosis, so obtaining a value for the “viable tumour” and avoiding partial volume effects. Of the previous studies investigating the prognostic ability of ADC values according to HPV OPC status, one study measured aROI^24^ whilst the other evaluated vROI.^4,13^ Our data indicate that the choice of ROI analysis may potentially influence the ability to predict survival outcomes.

A further aspect of the methodology in the current study was the acquisition of both ADC_mean_ and ADC_min_ parameters. Histological heterogeneity within tumour tissue may not be accurately reflected by the ADC_mean_. Whilst ADC histogram analysis is being explored,^10^ simple ADC parameters such as ADC_min_ may probe the different tumour cell populations. Since our vROI and aROI tumour contours were potentially influenced by partial volume effects from the air-filled lumen, the ADC_min_ was calculated as one standard deviation below the mean. However, this study did not demonstrate any benefit to using ADC_min_ in predicting treatment outcomes. Interestingly, Martens et al demonstrated the maximum ADC value to be a valuable prognostic pre-treatment parameter.^13^

There are potential criticisms concerning the study design, most of them fundamentally relating to erroneous assumptions prior to the power calculation. Whilst the sample size was comparable to other similar studies in the literature, the prospectively recruited study population comprised an unexpectedly high prevalence of HPV OPC (46/65). The dominance of the HPV OPC subgroup led to a smaller number of HNSCC participants which limited the scope of robust statistical comparisons with survival outcomes. Moreover, it is recognised that HPV OPC is associated with a lower rate of treatment failure than the 30% applied to the power calculation^2^ with a wider standard deviation of ADC values.^25^ This introduces the potential for Type 2 errors, thus also limiting the ability of pre-treatment ADC values to predict treatment failure in the HPV OPC subgroup. However, the similarity between ADC values in HPV OPC participants with and without 2 year LRFS and DFS (Tables 3 and 5) would argue that the inability to predict survival represents a true negative finding. Nonetheless, it is proposed that larger cohorts are required to adequately power future similar studies of both HPV OPC and other HNSCC participants.^22^ Finally, the assessment of interobserver agreement statistics was suboptimal since it was only performed in a proportion of the participants; however, this was representative of the whole sample with respect to tumour site and HPV status.

In conclusion, this comprehensive methodology adds to the recent data concerning the impact of HPV OPC status on the ability of pre-treatment ADC values to predict survival outcomes. The apparent association between lower pre-treatment primary tumour ADC_mean_ and 2 year LRFS, was not confirmed on multivariate analysis of all participants or in the subgroup analysis of the HPV OPC participants. A potential explanation for this is that the HPV OPC status, which is widely accepted to carry a favourable prognosis, also happens to demonstrate lower ADC_mean_ values. Larger cohorts with separate analyses of subgroups stratified by HPV-OPC status are required in order to advance our understanding of the optimal utilisation of DW-MRI in the evaluation of treatment responses in HNSCC.
